# Effects of two exercise programs on health-related fitness, quality of life and exercise enjoyment in adults with visual impairment: a randomized crossover trial

**DOI:** 10.1186/s13102-022-00566-3

**Published:** 2022-09-28

**Authors:** Rafael B. P. da Silva, Eduardo L Caputo, Natan Feter, Felipe F Reichert

**Affiliations:** 1 Postgraduate Program in Physical Education, Federal University of Pelotas, Rua Luís de Camões, 625, 96055630 Pelotas, Brazil; 2 School of Human Movement and Nutrition Sciences, The University of Queensland, 4067 St Lucia, QLD Australia

**Keywords:** Exercise, Visually impaired persons, Blindness, Quality of life

## Abstract

**Background:**

So far there is no study comparing two distinct exercise interventions in people with visual impairment. This study aimed to evaluate the effects of two exercise programs on health-related fitness, quality of life, and exercise enjoyment in people with visual impairment.

**Methods:**

Two exercise interventions were conducted: sports (i.e., Goalball, Football 5-a-side and Martial Arts) and functional training (i.e., Bodyweight exercises). Physical fitness was assessed by handgrip strength, flexibility, abdominal endurance, and cardiorespiratory fitness. The Physical Activity Enjoyment Scale (PACES) measured the pleasure in the practice of physical exercises, and the quality of life was measured by the WHOQOL-Bref.

**Results:**

A significant time x group interaction terms were observed for flexibility (*P* < 0.001; Cohen d = 0.08), abdominal muscular endurance (*P* < 0.001; Cohen d = 0.15), and distance covered in the 6-min walk/run test (*P* = 0.018; Cohen d = 0.02). An improvement of 13.3% and 5.1% on the distance covered on the 6-min walk/run test after sports and functional training was also observed.

**Conclusion:**

The functional training program was reported as more pleasant for participants. Sports-related exercises and functional training improved health-related fitness and quality of life for people with visual impairment.

## Background

The benefits of physical activity are observed throughout the lifespan, from childhood to elderly in people with and without disabilities [1]. However, a higher proportion of people with disability did not comply with physical activity recommendations (56.9%) when compared to people without disabilities (35.0%) [2]. Among those with disability, people with visual impairment (VI) are the most inactive. Steps per day and moderate-to-vigorous physical activity are reduced by up to 17% and 30%, in this population [3].

Difficulties for commuting, lack of public policies, discrimination, professionals not properly skilled to work with this population, fear of injury, lack of family support, and functional profile of VI (e.g., greater or lesser visual acuity, time living with VI, loss of central or peripheral vision) are among the main barriers to physical activity practice in people with VI [4, 5]. Furthermore, exercise practice may have important benefits in physical function and quality of life in this population. Sports-based programs might improve strength and balance [6, 7], and functional exercises programs can help in fall prevention [8], which is a main concern in this population. In addition, even though there is evidence on sports program benefits for people with disability, the literature is scarce regarding the impact of social and physical environment, as well as enjoyment [9].

In Brazil, there are about 4 million people living with VI [10], thus it is crucial to identify physical activities that promote health-related fitness, and are also enjoyable for these individuals which would increase their engagement in such activities. So far there is no study comparing two distinct exercise interventions in people with VI. Thus, the aim of this study was to evaluate the effects of two exercise programs on health-related fitness, quality of life and exercise enjoyment in adults with VI. One of the exercise programs was focused on sports-related exercises and the other on functional training.

## Methods

### Study design

This study was a randomized clinical trial (Brazilian Registry of Clinical Trials: BR-6yrddt), crossover design, 24 weeks long. There was an intervention period one (10 weeks); washout (4 weeks) and intervention period two (10 weeks). The washout period allows the dissipation of the first intervention effects before starting the second [11]. This design allowed us to compare two distinct exercise interventions and evaluate which of them had better outcomes.

Participants were randomly assigned in an exercise group of functional training (intervention period one), followed by sports games (intervention period two), or sports games in period one followed by functional training in period two. Health-related fitness, quality of life and enjoyment were assessed at baseline and after 24 weeks of intervention. This procedure was established according to the “CONSORT” statement for crossover design studies [12].

### Participants

Participants were recruited from the Louis Braille Association, a facility that provides services (e.g., rehabilitation and education programs) for people with VI in Pelotas, Rio Grande do Sul, Brazil. The Louis Braille Association is a reference institution for more than 200 people with VI from Pelotas and other cities in the state region.

Eligible participants were those aged 18 to 59 years, living in Pelotas and with a clinical diagnose of VI (i.e., those with visual acuity between 20/70 and 20/400, or worse than 20/400) [13]. To be considered eligible for the study, individuals should be able to perform physical exercises according to the Physical Activity Readiness Questionnaire (PAR-Q) scores. Those with other associated impairments (physical, hearing or intellectual) were not included.

### Sampling strategy and randomized allocation

Sample size calculation were based on cardiorespiratory fitness improvement using data provided elsewhere [14]. The minimum number of participants required to detect differences of ~ 5ml.kg-1.min-1 in the VO2max variable considering 80% of power and alpha of 0.05 was 12. In order to account for possible losses of follow-up and refusals, and to better manage the exercise sessions, we decide to double the required sample size. Thus, 24 out of the 53 eligible individuals of the institution were sampled. The groups were matched by gender (12 men and 12 women) and age (six men and women between 18–39 years old and six men and women between 40–59).

Based on a list provided by the institution, there were 53 eligible participants to be enrolled in the study. All members of this list were invited by phone, to take part in the study and 29 refused to participate. Those who accepted were matched by gender (12 men and 12 women) and age (six men and women between 18–39 and six men and women between 40 and 59). According to the ranking reached by the cardiorespiratory fitness tests, participants were allocated into two groups: odd numbers in a group and even numbers in the other. Afterwards, there was a draw to define which type of exercise the groups would perform first: functional training or sports-based exercises.

**Fig. 1:**
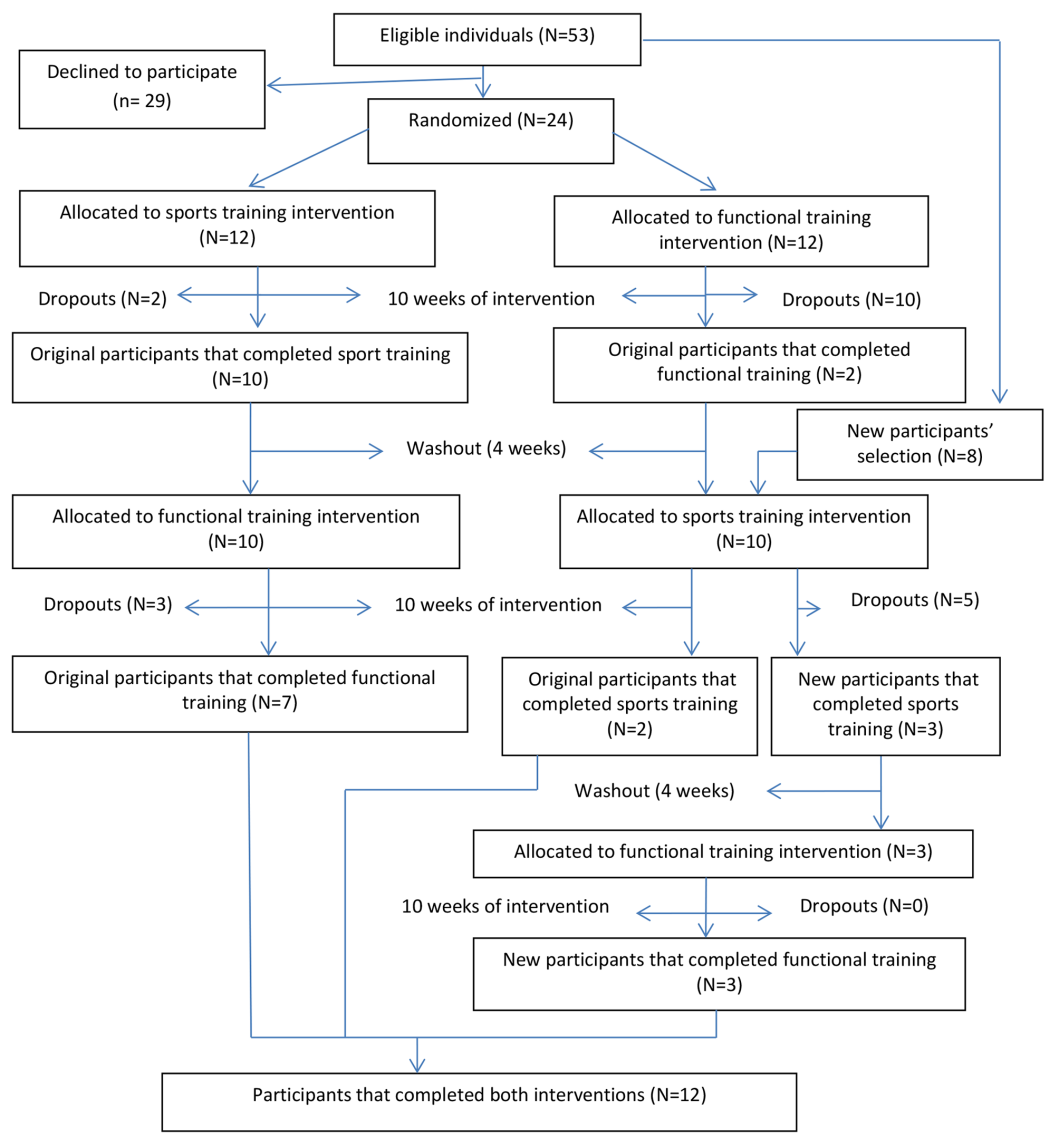
Flow diagram of subjects in the trial.

Throughout the first phase of the intervention, some participants dropped out and a new sample selection was carried out to keep the statistical power adequate for analyzes. At the end of the second phase, there was another washout period of four weeks followed by further 10 weeks of exercises sessions with those participants who entered the study at the later stage. Figure 1 describes the study’s sample flowchart.

### Physical exercises interventions

Both exercise programs were performed for 10 weeks. Exercise sessions were conducted three times per week (Tuesdays, Thursdays and Fridays), and lasted from 30 to 55 minutes. All exercises sessions were conducted at the Louis Braille Association yard (dimensions: 12 x 5m).

In order to make the exercise sessions more attractive, the sports-based exercises group was composed by three main activities: Goalball, Football 5-a-side and Martial Arts. These three activities were alternated throughout the intervention weeks. Also, activities aiming to develop motor and sensorial skills related to sports (e.g., activities necessary to develop ball hearing perception), as well as specific exercises of the sports modalities were performed.

Bodyweight exercises, such as sit-ups, squats and push-ups were performed in the functional training program. The sessions were organized in blocks of 10 exercises that were performed by the participants at the same time. The two exercise programs were similar regarding volume and intensity. A linear periodization of volume was used, as follows: at weeks 1 and 2, 30 min; week 3, 40 min; weeks 4 to 6, 45 min; weeks 7 to 9, 50 min; and week 10, 55 min. The Borg Ratings of Perceived Exertion (RPE) scale [15] was used to asses exercise intensity. In the first five weeks, participants should perform the exercise in a 3–5 (“moderate” to “strong” intensity) RPE, and in the next five weeks, between 6–9 ("more than strong" and "very, very strong" intensity).

### Health-related fitness

The following health-related fitness outcomes were assessed: cardiorespiratory fitness (adapted 6-min walk/run [6MWT] test), handgrip strength, flexibility (sit and reach test) and abdominal muscular endurance (maximum number of repetitions in one minute). Tests were conducted in consecutive days. The adapted 6MWT test was performed in the first day, while the other tests in the second day.

The adapted 6MWT was conducted in a public park near to the Louis Braille Association. Participants should run alongside a 20 m-rope, placed at the waist level, holding a tube (the rope passes through this tube), as many times as possible within 6 min. After the evaluator signal, participants began to run alongside the rope from one end to another, systematically changing the hand holding the tube at each turn. The evaluator warned the participant at minutes 2, 4 and 5.

The handgrip strength, flexibility and abdominal resistance tests were performed in the Louis Braille Association. A dynamometer (Takei Scientific Instruments, model T.K.K.5401) was used to assess the handgrip strength. Participants were sitting, with the elbow at 90º, the forearm in a neutral position and the wrist between 0° and 30°. Three attempts of maximum grip strength (15s rest x 3s isometric contraction) were performed and the highest value was considered.

A box (30.5 × 30.5 cm) was used to assess flexibility through the sit-and-reach test. Participants should be barefoot and put their feet against the box. The evaluator held the participant’s knees to prevent them from flexing. Participants should extend the arms with the palms down and flex the torso towards their feet. There were three attempts, and the highest value achieved was considered.

To assess abdominal muscular endurance, participants were in supine position with the knees flexed at 90º and their feet in total contact with the ground. Participants should raise the trunk until it touched the knees, returning to the starting position. Each touch of the trunk on the knees completes a flexion. Participants should perform the maximum number of possible repetitions in 60 s.

Between the three tests, there was an interval of 3 to 5 min of passive recovery. The tests were applied with appropriate adaptations for individuals with VI (e.g., to those who were blind, rather than demonstration by mime, the correct executions of the tests were showed by touch).

### Quality of life

The World Health Organization Quality of Life questionnaire (WHOQOL-Bref) was used to assess quality of life. Data were analyzed in each domain separately (Physical Health, Psychologic, Social Relationships and Environment) and in its total score. Quality of Life self-perception and satisfaction with health were evaluated by questions 1 and 2 of the WHOQOL-Bref, respectively.

### Enjoyment of the physical exercise practice

The Portuguese version of the Physical Activity Enjoyment Scale (PACES) was used to assess the level of physical exercise enjoyment. Each of the eight PACES questions has a Likert scale (from 1 to 7, which 1 could be “*It’s no fun at all*” and 7, “*It’s a lot of fun*”, for example). Data were analyzed by total score and by each question score. Further, participants were questioned: *“From the two types of exercises that were practiced which one did you like the most?* The possible answers were: *a) Sports; b) Functional training; c) I enjoyed both equally; d) I did not like either.“*

### Statistical analysis

Data were double-typed in an Excel spreadsheet and transferred for statistical analysis in Stata/IC14.1.

All outcomes were described by means and standard deviation (SD). The Shapiro-Wilk test was used to check for normality. Health-related fitness and quality of life outcomes were analyzed by two-way ANOVA with repeated measures, and Bonferroni’s post hoc was applied as required. According to the assumptions for its use, t-test or Wilcoxon test were used for the PACES variables. All tests were done at P < 0.05.

## Results

Figure 1 shows the participants flow through the trial. Of the 53 eligible individuals, 24 were initially randomized (Sports:12; Functional:12). The main reasons for dropping out were: priority to other activities *[n = 3]*, financial restraints *[n = 2]*, health issues *[n = 5]*, lack of motivation *[n = 6]*, commuting issues *[n = 1]*, and reason not reported *[n = 3]*. Initially, 15 participants dropped out (5 from the sports group and 10 from the functional training group). Afterwards, eight new participants were included into functional training group, and further 5 dropped out.

Table 1 shows the descriptive characteristics of participants. Twelve participants (seven men) aged 30 to 59 (49.1 ± 8.7), 50% of them were blind, and the mean time living with VI was 26.8 years (95%CI 13.8; 39.7). Most of participants were white (58.4%) and acquired their VI (58.3%) (Table 1). Mean intervention adherence was 77.1% (SD = 20.2) and 66.4% (SD = 21.7), in sports and functional training groups (*P* = 0.1), respectively.

**Table 1:** Descriptive characteristics of participants (n = 12)

	Mean ± SD
Age (years)	49.1 ± 8.7
Weight (Kg)	76.1 ± 13.9
Height (m)	1.7 ± 0.1
Time living with visual impairment (years)	26.8 ± 20.4
	N (%)
Gender	
Male	7 (58.3)
Female	5 (41.7)
Skin color	
White	7 (58.4)
“Pardo”/black	5 (42.6)
visual impairment	
Acquired	7 (58.3)
Born	5 (41.7)

Data regarding the effects of both interventions in health-related physical fitness and quality of life outcomes are shown in Table 2. There were significant time x group interaction terms for flexibility (*P* < 0.001; Cohen d = 0.08), abdominal muscular endurance (*P* < 0.001; Cohen d = 0.15), and distance covered in the 6MWT (*P* = 0.018; Cohen d = 0.02). Abdominal endurance increased 31.6% and 9.4% after sports and functional training, respectively. Also, the distance covered in the 6MWT was improved by 13.3% and 5.1% after sports and functional training, respectively. Regarding the quality of life, an improvement in physical domain was observed after the functional training program (7.7%), and in the environmental domain after sports training (7.5%).

**Table 2:** Participants physical fitness pre- and post-intervention (N = 12)

	Sports		Functional training		p-value Time	p-value Group		p-value Time x group
	Baseline	Post-intervention	Baseline	Post-intervention			Cohen d**	
**Physical fitness**								
*Handgrip strength (kg)*	33.2 (9.8)	33.5 (10.1)	34.0 (10.7)	34.7 (10.5)	0.449	0.781	0.12	0.860
*Flexibility (cm)*	24.4 (13.7)	22.3 (12.5)*	23.8 (13.5)	23.3 (12.3)	0.142	< 0.001	0.08	< 0.001
*Abdominal muscular endurance (rep in 1 min)*	15.2 (13.0)	20.0 (15.1)*	20.2 (13.0)	22.1 (13.1)	< 0.001	0.001	0.15	< 0.001
*Distance covered in 6-min walk/run test (m)*	563.3 (167.5)	638.3 (153.6)*	605.4 (154.6)	636.3 (152.2)*	< 0.001	0.533	0.02	0.018
**Quality of life domain**								
*Physical*	26.0 (4.9)	26.6 (4.4)	26.0 (4.9)	28.0 (4.2)*	0.633	0.002	0.33	0.226
*Psychologic*	23.2 (3.5)	24.6 (3.0)	24.5 (3.2)	24.6 (3.9)	0.025	0.369	0.00	0.159
*Social relationships*	12.2 (2.7)	12.5 (2.5)	12.3 (3.0)	12.5 (2.6)	0.802	0.698	0.00	0.723
*Environmental*	28.0 (5.1)	30.1(5.1)*	31.0 (5.9)	30.7 (6.0)*	0.147	0.048	0.11	0.107
*Self-perception of health*	4.1 (0.9)	4.1 (0.8)	4.1 (0.8)	4.1 (0.8)				
*Satisfaction with health*	3.6 (1.2)	3.8 (1.0)	3.6 (1.3)	3.8 (1.1)				
*Total score*	97.1 (15.5)	101.6 (10.6)	101.5 (17.0)	103.7 (15.2)	0.744	0.025	0.16	0.402

*P < 0.05 compared to baseline. **between groups post-intervention

Table 3 shows the PACES results. Functional training was described as more pleasant (*P* = 0.047) and obtained a greater total score (*P* = 0.031) than sports. Regarding the specific question which compared the intervention enjoyment, 25% of the participants reported enjoying more the sports classes, 25% enjoyed more the functional training classes and the other half both interventions equally.

**Table 3:** PACER’s scores after the two interventions (N = 12)

PACER categories	Sports	Functional training	
	Mean (SD)	Mean (SD)	*p*
Pleasurable	6.3 (1.1)	6.8 (0.6)	0.236*
Fun	6.3 (1.0)	6.8 (0.6)	0.053*
Pleasant	6.2 (0.9)	6.9 (0.3)	0.047*
Invigorant	6.2 (1.2)	6.8 (0.6)	0.131*
Gratifying	6.5 (0.8)	6.9 (0.3)	0.096*
Exhilarating	6.8 (0.6)	6.9 (0.3)	0.438*
Stimulating	6.6 (0.7)	6.7 (0.6)	0.723*
Refreshing	5.7 (1.5)	5.9 (1.6)	0.463*
Total score	50.6 (5.4)	53.7 (3.1)	0.031**

*T test

**Wilcoxon test

## Discussion

Our study showed that exercise interventions in people with VI were beneficial for abdominal muscular endurance and cardiorespiratory fitness. Also, exercise interventions showed important effects on Physical and Environmental quality of life domains. Despite positive effects on health observed from both exercise programs, functional training was considered more pleasant than sports-based exercises. To our knowledge, this was the first study to explore the effects of different physical exercise programs on health-related outcomes in people with VI.

Regular physical activity/exercise practice is important to improve or maintain health-related fitness, which includes aerobic capacity, muscular fitness, flexibility, speed and balance [16]. People with VI usually have more barriers to participate in regular exercise programs, as some barriers are related to their disability. Also, most studies applied only one type of exercise intervention, which limits the interpretation of their effects on fitness variables [17–20]. Our study applied two different exercise interventions comprising a wide range of body movements, expanding the benefits for health-related physical fitness, as it was not designed to improve one specific health-related outcome.

Although an increased aerobic capacity was observed after both interventions, it was more pronounced after the sports program (13.3%). In the sports activities selected (goalball, football 5-a-side and martial arts), participants spent more time running and walking, when compared to functional training activities, which may have contributed for this result. Thus, an improved aerobic fitness assessed by the 6MWT might improve the ability of people with VI to perform daily living activities more easily, especially those related to active commuting and walking [21].

There is a lack of literature regarding exercise interventions aiming to improve physical fitness in people with VI, as previously stated in the introduction. A recent meta-analysis showed no combined effects of exercise interventions for balance and functional capacity in this population [22]. Our data indicated an increase in abdominal muscle endurance and cardiorespiratory fitness, which may help people with VI performing their daily tasks. In addition, higher cardiorespiratory and muscle fitness are associated with a reduced risk for several chronic diseases and all-cause mortality [23].

We recently showed that people with VI are more likely to report improved quality of life when engaging in sufficient levels of physical activity [24]. However, evidence regarding physical activity and quality of life outcomes in people with VI is mostly from observational studies, and lacks on evaluate a specific type of activity, such as sports or non-sports activities [24]. Our data supports the hypothesis of a causal relationship between exercise practice and increased quality of life, particularly through the physical and environmental domains.

When compared to people without disabilities, a higher probability to report chronic conditions (e.g., heart disease, diabetes) is observed among people with disabilities. The higher prevalence of physical inactivity in this population likely contributes to this increased risk of diseases [25]. The mobility restrictions (e.g., blocked or slippery footpaths) and lack of suitable conditions [26] contributes to physical inactivity in this population, which increase the risk of developing several chronic diseases. Engaging in physical activity decreased activity limitations and increased the ability to independently perform daily usual tasks in this population [27]. Our study showed that a structured exercise program can have an important impact in the physical quality of life domain in people with VI.

One should note that the environmental domain accounts for almost one-third of the WHOQOL-Brief (eight out of the 26 questions) and contains aspects that are not related to the individual or interventions, such as perception of safety and environmental characteristics (e.g., climate, pollution, noise). The intervention must impact the physical, psychological and social domains significantly more than the environmental domain, since such domain is unlikely to change solely with an exercise program. Although exercise programs somehow improved the psychological and social relationship domains [28, 29], a longer intervention period might be necessary for such changes to take place.

Health improvements and social interaction are some of the factors that motivate people with disabilities to engage in physical exercise programs [29]. Similarly, physical exercise practice is strongly associated to the pleasure in performing activities [30]. Thus, individuals who enjoy exercising are much more likely to be physically active [31]. According to the data obtained by PACES, participants reported that functional training was more pleasant when compared to sports activities.

Although sports activities have characteristics such as playfulness and competition, which are associated with motivation and pleasure [30], it is possible that a lack of self-efficacy in performing these activities impacted participants’ perception [32]. In sports-based exercises, some activities have a greater complexity of execution compared to the functional training exercises (e.g., kicking a ball in a specific direction requires greater motor skills than squatting). In addition, only in sports classes (i.e., goalball and football 5-a-side) some exercises were practiced with all participants blindfolded. In these exercises, the participants with low vision might have had a greater fear of injury by the deprivation of their residual vision, therefore decreasing exercise practice enjoyment.

The limitations of our study should be pointed out. First, exercise intensity was controlled by RPE, and all participants were familiar with this scale in order to pinpoint the exercise intensity with precision. However, we understand that a lack of an objective intensity measure might be a limitation of our study. Heart rate, for instance, despite being an objective measure could not be used in this study context, given the distinct nature of the exercises in the two programs. Second, sampling selection bias cannot be ruled out, because all participants were selected from the same institution. Since the Louis Braille institution is the only one specialized for people with VI in the city, it is possible that those not attending the institution may have different characteristics which could compromise external validity. Finally, there was a high rate of dropouts and this could have two consequences: a) lack of adequate statistical power and b) selection bias. To address this issue, another group of participants were selected, thus statistical power remained at the required levels. In addition, comparison between participants in each group were similar at the baseline regarding relevant characteristics, thus we do not believe that the rate of dropouts has affected our results. Thus, future studies with larger and representative samples are important to compare with the data presented here.

This study also has methodological aspects that must be highlighted. It is important to note that most of the studies with this population are conducted in high-income countries. However, 90% of global VI is located in medium-/low-income countries [33], such as Brazil, reinforcing the relevance of our study. Another important aspect is the crossover design, which increases the statistical power, making it possible to test hypotheses with a smaller number of participants. The exercises used in this study can be easily performed with few materials (e.g., mats, balls, cones). Thus, one can replicate/adapt the programs according to their conditions, facilities and professionals.

## Conclusion

Sports-related exercises and functional training improved health-related fitness and quality of life for people with VI. Exercise interventions showed positive effects in cardiorespiratory fitness, abdominal endurance, as well as the physical and environmental domains of quality of life. Also, functional training exercises were considered more enjoyable.

## Data Availability

The datasets used and/or analysed during the current study are available from the corresponding author on reasonable request.
